# Next-Generation Joint-on-a-Chip: Toward Precision Mechanical Control in Multi-Tissue Systems

**DOI:** 10.1007/s40820-025-02031-5

**Published:** 2026-01-05

**Authors:** Zhenjun Lv, Yuwei Chai, Xiumei Zhang, Weiwei Lan, Junchao Wei, Lu Li, Weiyi Chen, Yiting Lei, Jun Liu, Zhong Alan Li, Di Huang

**Affiliations:** 1 Department of Biomedical Engineering, Research Center for Nano-Biomaterials and Regenerative Medicine, Shanxi Key Laboratory of Functional Proteins, College of Artificial Intelligence, Taiyuan University of Technology, Taiyuan, 030024 People’s Republic of China; 2 Institute of Biomedical Engineering, Shanxi Key Laboratory of Materials Strength and Structural Impact, Taiyuan University of Technology, Taiyuan, 030024 People’s Republic of China; 3 Shanxi Key Laboratory of Bone and Soft Tissue Injury Repair, Second Hospital of Shanxi Medical University, Taiyuan, 030024 People’s Republic of China; 4 Department of Biomedical Engineering, Faculty of Engineering, The Chinese University of Hong Kong, Shatin, N.T., Hong Kong SAR, People’s Republic of China; 5 Peter Hung Pain Research Institute, Faculty of Medicine, The Chinese University of Hong Kong, Shatin, N.T., Hong Kong SAR, People’s Republic of China

**Keywords:** Joint-on-a-chip, Osteoarthritis, Tissue microenvironment, Mechanical stimulation, Multi-tissue co-culture

## Abstract

Osteoarthritis is among the leading causes of disability worldwide, and no pharmacological therapies currently exist to reverse its progression. This lack of therapies is primarily attributed to the inadequacies of conventional in vitro models of joint physiology and pathology, which significantly hinder advancements in disease mechanism research and drug development. As an emerging in vitro joint model, joint-on-a-chip (JoC) technology allows low-cost, efficient simulation of physiological and pathological joint activities, making it a focal point of current research. Cartilage, subchondral bone, and synovium are among the key tissues required for constructing in vitro joint models, with cartilage playing a central load-bearing role in joint movement. This article provides a detailed overview of the structure and function of these tissues, with an emphasis on the load-bearing mechanisms of cartilage, and identifies the microenvironmental characteristics that JoC should aim to replicate. Subsequently, we review the current types of JoC and highlight their core challenge: the seamless integration of multi-tissue co-culture with specific mechanical stimulation. To address this issue, we propose potential solutions and present a conceptual design for a JoC prototype. Finally, we discuss the challenges and issues related to the outlook for JoC. Our ultimate goal is to develop a JoC capable of replicating the key microenvironments of joints, serving as a high-performance in vitro joint model to advance the study of disease mechanisms and facilitate drug development.

## Highlights

- Outlines key structural and microenvironmental features of joints.
- Discusses strategies to integrate mechanical stimulation with multi-tissue co-culture.
- Proposes innovative design concepts toward next-generation joint-on-a-chip platforms.

## Graphical abstract

**Figure:**
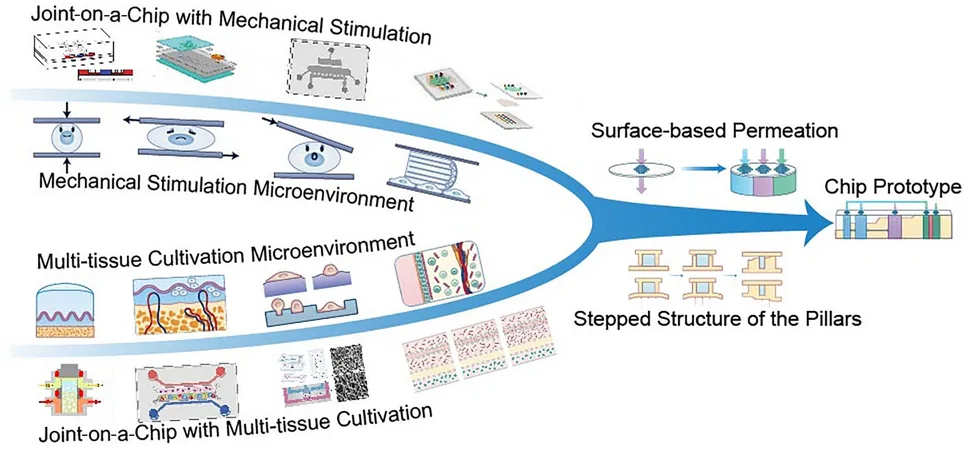

## Introduction

Osteoarthritis (OA) is the most prevalent form of arthritis, a degenerative joint disease, and the leading cause of physical disability in older adults. As a degenerative disorder, it affected approximately 595 million people worldwide in 2020, corresponding to 7.6 percent of the global population [1]. This disease not only causes severe pain for patients but also imposes a significant socioeconomic burden. Currently, no drug exists to halt or reverse the progression of OA due to its complex etiology, which involves multiple factors such as aging, obesity, trauma, and abnormal mechanical loading [2–4]. The underlying pathogenic mechanisms are thought to result from the interplay of mechanical, cellular, and inflammatory factors. OA affects the entire joint, with pathological changes impacting articular cartilage, subchondral bone, synovium, ligaments, menisci, and joint capsule. Current research suggests that abnormal mechanical stimuli initially damage these tissues, inducing the release of extracellular mediators and activating inflammatory pathways, thereby driving disease progression [5].

The heterogeneity of OA pathogenesis makes it difficult to create in vitro models that truly replicate human joint physiology, posing a major obstacle to drug discovery. To overcome this challenge, researchers employ a spectrum of OA models—ranging from two- (2D) and three-dimensional (3D) cell cultures to small and large animals—to probe disease mechanisms and screen candidate therapeutics [6, 7]. Each model, however, has specific drawbacks. Conventional 2D/3D cultures lack extracellular matrix (ECM) signaling, fluid shear, concentration gradients, and mechanical stimulation, so they cannot fully reproduce the joint microenvironment [8]. Dedicated loading platforms (e.g., compressive rigs, uniaxial stretchers, FlexCell systems) add mechanical cues but remain expensive, labor-intensive, and only partially biomimetic [9]. Animal models suffer from interspecies differences, long study times, high costs, and ethical concerns, which limit their translational value [10, 11]. Consequently, existing approaches still fall short of delivering an in vitro joint or OA model that integrates multiple tissues and complex mechanical stimuli. There is therefore an urgent need for a cost-effective, physiologically relevant in vitro joint model capable of precisely mimicking the joint-specific microenvironment.

Organ-on-a-chip (OoC), also known as microphysiological systems, integrates microfluidics and tissue engineering technologies to construct miniaturized in vitro tissue culture platforms that recreate key functions of human organs [12]. Since the groundbreaking development of the lung-on-a-chip [13, 14], successful models have been established for the gastrointestinal tract [15], liver [16], kidney [17], pancreas [18], heart [19], and vasculature [20], demonstrating superior biomimetic performance. Similarly, a joint-on-a-chip (JoC) can facilitate 3D co-culture of multiple tissues on a single platform while simultaneously applying precise mechanical stimuli to recreate the overall joint microenvironment in vitro [21]. Its low-cost, high-throughput, and operational simplicity are anticipated to make it a vital tool for future OA research.

The joint is a multi-tissue system primarily composed of articular cartilage, subchondral bone, synovium, and ligaments, with weight-bearing joints such as the knee also including the meniscus. These tissues, together with the infrapatellar fat pad (IPFP), muscles, tendons, and the patella, collectively maintain joint homeostasis, with mechanical stimuli serving as a key regulatory factor for their normal function [22, 23]. To balance experimental complexity with physiological relevance, a JoC should at minimum integrate cartilage, subchondral bone, and synovium while replicating mechanical stimuli closely associated with joint homeostasis. Several reviews have already discussed JoC models developed for different tissues; however, these studies have primarily focused on the biological components [21, 24] and application aspects of the chips [24]. In contrast, the present review emphasizes the design of physiologically relevant JoC systems, exploring how rational engineering design can best recapitulate the complex joint microenvironment characterized by multi-tissue crosstalk and mechanical stimulation. This review first provides an overview of the structure and function of these three important tissues, with an emphasis on the mechanical characteristics and microenvironment of cartilage, as cartilage degradation is a central feature of OA pathology [21, 24]. Subsequently, it reviews existing types of JoC, analyzes their limitations, and proposes design requirements for an ideal JoC platform (Table 1). Notably, other joint tissues—such as the IPFP and the menisci—also play significant roles in OA and other joint disorders. However, because JoC models that incorporate these tissues are still scarce, they are not examined in detail herein. Finally, it outlines feasible technical approaches to address key challenges in combining multi-tissue co-culture with specific mechanical stimuli.

**Table 1:** Detailed data on the simulation of microenvironments on different JoC

	Cartilage			Subchondral bone				Synovium		
	Matrix	Compression	Shear	Matrix	Compression	Cell type	Culture environment	Shear	Cell type	Culture environment
Lee [78]	Alginate hydrogel	Non-uniform	N/A	N/A				N/A		
Paggi [79]	Agarose hydrogel	Multiaxial (non-quantified)	Multiaxial (non-quantified)	N/A				N/A		
Occhetta [82]	PEG hydrogel	Constrained uniform (quantified)	N/A	N/A				N/A		
Lin [86]	GelMA	N/A	Fluid shear	GelMA	N/A	Osteoblasts	Osteogenic medium	N/A	Fibroblasts	Fibroblast medium
Tuerlings [87]	N/A	N/A	N/A	PCL scaffold	N/A	Osteoblasts	Osteogenic medium	N/A		
Salehi [88]	Fibrin hydrogel	N/A	N/A	Fibrin hydrogel (with calcium phosphate)	N/A	Osteoblasts, osteoclasts, endothelial cells, and mesenchymal stem cells	Osteogenic medium/Osteoclast medium	N/A		
BAO [89]	N/A	N/A	Fluid shear	N/A	N/A	Osteoinduced mesenchymal stem cells	Osteogenic medium	N/A		
Mondadori [90]	Fibrin hydrogel	N/A	N/A	N/A				Fluid shear	Fibroblasts, endothelial cells, macrophages	Endothelial cell growth medium-2

## Joint Tissues and Their Microenvironments

Articular cartilage is an avascular tissue primarily composed of ECM and a small population of chondrocytes [25]. The ECM is rich in collagen, proteoglycans, and water, enabling cartilage to withstand compressive, tensile, and shear forces. The fibrous structure of the ECM is critical for resisting tensile and shear stresses. In the superficial zone, type II collagen fibers are arranged tangentially to dissipate shear and tensile loads, and proteoglycan-4 is secreted to lubricate the joint [26]. In the middle zone, fibers are randomly oriented to resist multidirectional forces, with compressive performance dependent on the relative displacement between fluid and solid components within the ECM. In the deep zone, fibers are arranged perpendicularly, and proteoglycan content is higher, aiding in water retention and compression resistance [27]. The interaction between type II collagen and proteoglycans/glycosaminoglycans imparts a negative charge to the ECM, attracting water and conferring compressive resistance and low frictional properties [28]. During loading, the ECM restricts fluid efflux and generates hydrostatic pressure; once the load is removed, interstitial water rapidly re-enters, allowing the tissue to recover its shape [29]. Additionally, high-frequency cyclic loading—exemplified by walking—and sustained loading can elicit dynamic fluctuations in tissue osmotic pressure lasting from seconds to hours. Such osmotic pressure fluctuations have been shown to modulate the chondrogenic transcription factor Sox9, thereby promoting or suppressing ECM synthesis [30, 31].

**Fig. 1:**
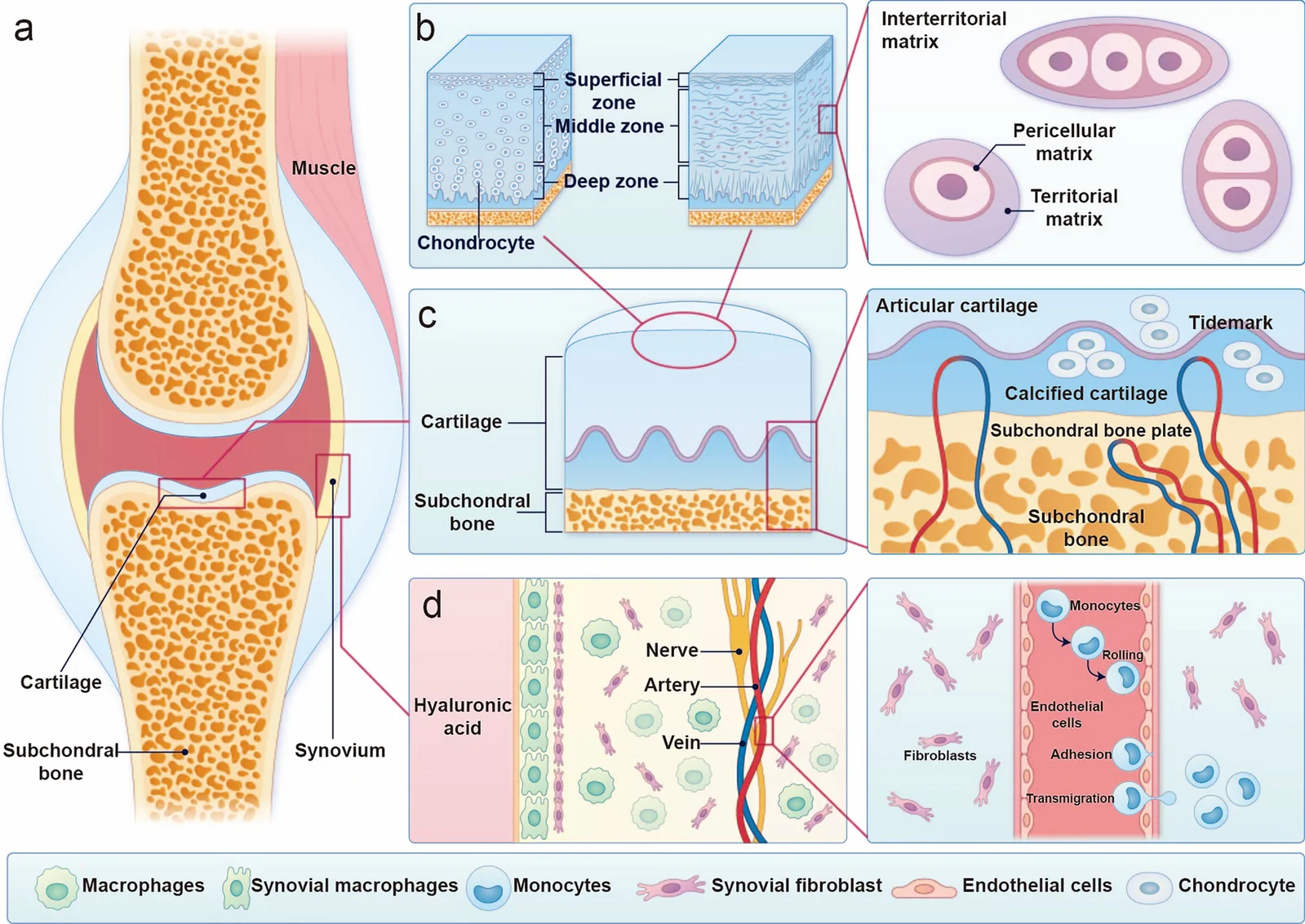
Schematic illustration of the functional structures of articular cartilage, subchondral bone, and synovium. **a** Overall anatomical schematic of a joint. **b** Diagram of the zonal organization of articular cartilage: the left side depicts the morphology and distribution of chondrocytes across different zones, while the right side illustrates the arrangement and orientation of collagen fibers. The magnified view highlights the structural characteristics of single chondrocyte unit and isogenous group. **c** Structural schematic of cartilage and subchondral bone. The magnified view on the right details the hierarchical structure of the subchondral bone, comprising, from superficial to deep layers, the tidemark, calcified cartilage, subchondral bone plate, and subchondral trabecular bone. **d** Schematic illustration of the synovium. The synovium consists of the lining layer and the sublining layer, which contain synovial fibroblasts, synovial macrophages, an abundant microvascular network, and nerve fibers. The magnified view on the right shows the detailed architecture of the microvasculature within the synovium

Chondrocytes exhibit morphological differences across zones: flattened in the superficial zone, oval in the middle zone, and spherical in the deep zone, reflecting their adaptation to mechanical stimuli (Fig. 1a). Each chondrocyte is encapsulated by a pericellular matrix (PCM), together forming the “chondrocyte unit.” The PCM, with an elastic modulus lower than that of the ECM, is rich in type VI collagen and modulates biomechanical and biochemical signal transduction (Fig. 1a) [32]. It protects superficial chondrocytes and may amplify local strain in the deep zone [33]. The development, degeneration, and regeneration of articular cartilage are all finely regulated by biomechanical cues. Cartilage primarily experiences four types of mechanical stimuli: compressive stress, fluid shear stress, hydrostatic pressure, and osmotic pressure. These stimuli can act independently or in combination [34]. Within physiological ranges, they maintain tissue homeostasis; when exceeding those ranges, they trigger pathological changes. Compression is the principal loading mode of cartilage. Moderate dynamic compression promotes ECM synthesis, whereas prolonged static compression suppresses the production of collagen and proteoglycans. Supraphysiological compression can induce cellular phenotypic alterations resembling those observed in OA [34]. Under compression, interstitial fluid is expelled, generating fluid flow and shear forces. Moderate shear stress enhances the mechanical properties of cartilage, whereas excessive shear can damage cells and provoke inflammatory responses [35]. When fluid movement is restricted during compression, hydrostatic pressure (HP) develops; moderate HP (5–10 MPa) promotes ECM synthesis, but excessive HP may contribute to OA pathogenesis [36]. Alternating loading and unloading cycles cause fluctuations in osmotic pressure—moderate fluctuations support the functional maintenance of engineered cartilage tissues, whereas excessive fluctuations can impair cell viability [34].

Chondrocytes sense mechanical cues primarily through two mechanisms: (1) direct perception of PCM or ECM deformation, with mechanical signals transmitted via adhesion complexes and the cytoskeleton; and (2) indirect signaling mediated by mechanically induced release of growth factors that act through receptor pathways [5]. These mechanisms play essential roles in both cartilage homeostasis and degeneration. Recently, protein C receptor (Procr^+^) progenitor cells identified in the superficial zones of tibial articular cartilage and the meniscus have been shown to sense mechanical stress through the mechanosensitive channel Piezo1, thereby regulating cartilage regeneration. Appropriate mechanical loading increases the population of these cells, whereas under OA conditions, Procr⁺ cells are activated to repair damaged tissue [37]. Overall, chondrocytes maintain ECM homeostasis by sensing and responding to mechanical stimuli; however, chronic or excessive loading disrupts signaling homeostasis, leading to aberrant cellular phenotypes and pathological ECM remodeling.

The subchondral bone comprises inorganic components (hydroxyapatite) for stiffness and organic components (type I collagen, proteoglycans, glycosaminoglycans, and water) for elasticity [38]. It is divided into the subchondral bone plate (SBP) and trabecular bone (Fig. 1b). The SBP lies beneath the calcified cartilage and extends into the deeper trabecular bone, which distributes joint loads and protects cartilage. Stress is transmitted through the calcified cartilage to the subchondral bone, reducing shear stress [39]. The structure of trabecular bone varies with its proximity to the articular surface, adapting to the local mechanical environment [40]. Although the mechanical strain experienced by the subchondral bone is significantly lower than that in cartilage, osteocytes can still regulate bone remodeling through mechanosensory mechanisms [41, 42]. Subchondral bone remodeling depends on the dynamic balance between osteoclastic bone resorption and osteoblastic bone formation, processes that are mutually regulated via the RANK/RANKL/OPG signaling pathway [43, 44]. Osteocytes, embedded within the mineralized matrix, sense shear stress generated by fluid flow through the lacunar–canalicular system, thereby modulating the activities of osteoblasts and osteoclasts to initiate reparative responses. Under mechanical stimulation, the mechanosensitive channel PIEZO1 in osteoblasts can activate the YAP1 signaling pathway, which promotes the expression of COL2α1 and COL9α2 [45]. These collagen subtypes, in turn, negatively regulate osteoclast differentiation, forming a feedback regulatory loop.

In OA, the subchondral bone exhibits an increased bone turnover rate, accompanied by vascular invasion across the tidemark into the cartilage [46]. Abnormal mechanical loading is considered a primary trigger for the formation of type H vessels and bone marrow lesions. Such loading can alter local blood supply, modulate the release of growth factors, and activate mechanotransduction pathways—including Wnt/β-catenin, TGF-β/BMPs, and SDF-1/CXCR4—thereby promoting pathological remodeling and angiogenesis in the subchondral bone [43]. During OA progression, subchondral bone displays pathological alterations—including accelerated bone turnover, microstructural abnormalities, hyperactivation of transforming growth factor-β (TGF-β) signaling, angiogenesis, and aberrant sensory innervation—that individually or synergistically drive disease advancement [44, 47]. Angiogenic vessels invade the cartilage from the deep (calcified) zone toward the articular surface, inducing ectopic intrachondral ossification; meanwhile, abnormal sensory innervation is a principal mediator of OA-associated pain [48]. For example, calcitonin gene-related peptide (CGRP)–immunopositive sensory fibers within osteochondral plate channels have been implicated as key contributors to OA pain in both humans and rodent models [49].

The synovium consists of a lining layer (comprising synovial fibroblasts and macrophages) and a sublining layer (containing fibroblasts, macrophages, blood vessels, and nerves), which functions as both a filter and a barrier while contributing to joint lubrication and nutritional support (Fig. 1c) [50]. Synovial fibroblasts secrete lubricin and hyaluronic acid, reducing cartilage wear, whereas macrophages clear debris and regulate inflammation and tissue repair [51, 52]. Under physiological conditions, the synovium is subjected to cyclic tensile strain during joint flexion and extension [53]. A static stretch of 10% strain upregulates hyaluronan synthase 2 (HAS2) mRNA expression, thereby promoting hyaluronic acid synthesis [54]. Low-frequency tensile strain is relatively well tolerated by OA synovium and is associated with enrichment of pathways related to interferon response, Fc receptor signaling, and lysosomal transport, suggesting activation of inflammation-resolving mechanisms [55]. In contrast, high-frequency strain increases the production of lactate and 3-nitrotyrosine (3-NT), while activating NOD-like receptor and neutrophil degranulation pro-inflammatory pathways, indicating that high-frequency mechanical stimulation tends to shift the tissue toward a pro-inflammatory state [53]. Patients with late-stage knee OA often exhibit acute intolerance to high-frequency physical activities such as brisk walking, suggesting that OA-induced pathological changes disrupt the mechanical homeostasis of the synovium and diminish its tolerance to mechanical loading. In summary, the synovial response to mechanical loading is dual in nature: under physiological conditions, it facilitates lubrication and tissue repair, whereas under OA conditions, it can amplify inflammation and exacerbate pain [56].

Although OA is not generally regarded as an inflammatory arthropathy, focal synovitis is common in OA [57]. Histologically, synovitis is characterized by thickening of the synovial lining, increased vascular density, and inflammatory cell infiltration; among these changes, accumulation of synovial macrophages plays a pivotal role in cartilage-matrix degradation [57, 58]. Single-cell RNA-seq (scRNA-seq) has revealed marked cellular heterogeneity, with the following populations ranked by abundance: sublining fibroblasts, lining fibroblasts, HLA-DRA + cells—comprising immunomodulatory and inflammatory macrophages, dendritic cells, activated pro-inflammatory HLA-DRA + fibroblasts, and B-cell subsets—smooth muscle cells, endothelial cells, T cells, mast cells, and proliferative immune cells [59]. Synovitis also markedly reduces nerve fiber density in the synovial lining and, via fibroblast activation, sensitizes nociceptive fibers, thereby intensifying pain [60, 61]. Consequently, analgesia remains a core objective of ongoing OA drug development programs.

**Fig. 2:**
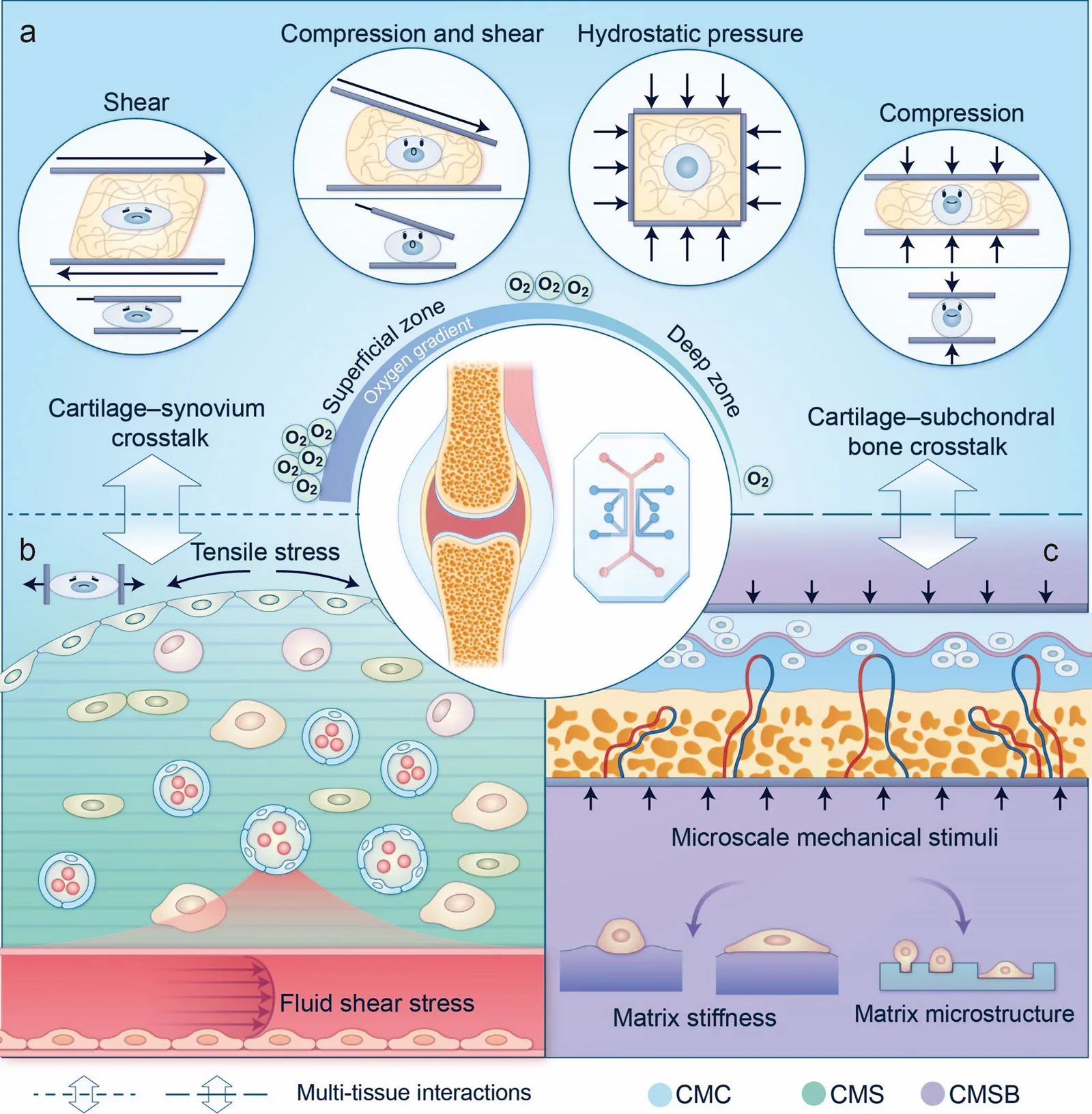
Schematic illustration of key joint microenvironments to be recapitulated in JoC systems. **a** The cartilage microenvironment, which includes mechanical stimuli such as shear stress, multiaxial compressive stress, uniaxial compressive stress, and pervasive hydrostatic pressure experienced by the superficial, middle, and deep zones of cartilage during joint movement. Additionally, the chemical microenvironment of cartilage (CMC, indicated by the blue background) and oxygen gradient must also be simulated. **b** The synovial microenvironment is characterized by various types of synovial cells, the chemical microenvironment of synovial (CMS, indicated by the green background), and mechanical forces—specifically tensile stress of the synovial lining and intravascular fluid shear stress. **c** The subchondral bone microenvironment, featuring the chemical microenvironment of subchondral bone (CMSB, indicated by the purple background), microscale mechanical stimuli, resident cell populations, specialized microstructural characteristics, and surface stiffness. Moreover, there is complex inter-tissue signaling among cartilage, subchondral bone, and synovium, represented by different line styles: dashed lines of varying thickness indicate different strengths of interaction, while solid lines indicate the absence of direct communication

Constructing a JoC requires the faithful recreation of the microenvironments of joint-associated tissues. As the principal tissues that sustain joint homeostasis and drive OA progression, the microenvironments of cartilage, subchondral bone, and synovium must therefore be rigorously defined, thereby providing a sound theoretical basis for recapitulating joint architecture and function on-chip (Fig. 2).

First, mechanical stimuli are indispensable elements within the microenvironment of these three tissue types. Cartilage is continuously subjected to multiaxial stresses, including compressive and shear forces (Fig. 2a, b). Subchondral bone, due to its high elastic modulus, undergoes only minimal strain, which allows osteocytes to regulate their physiological activities accordingly [62]. Chondrocytes are additionally exposed to relatively uniform hydrostatic pressure, while the synovium is subjected mainly to tensile stress during joint motion. In vitro studies show that multiaxial stress and hydrostatic pressure induce chondrocytes to adopt physiologically relevant and osteoarthritic phenotypes, respectively [63]. Similarly, tensile forces experienced by the synovium have been shown to closely correlate with hyaluronic acid secretion and the progression of OA [53, 64].

Second, the architectural characteristics of the three tissues critically influence their function. Cartilage possesses a stratified, load-bearing architecture; its heterogeneous mechanical stimuli should therefore be accurately mimicked on-chip. Extensive evidence demonstrates that matrix organization, surface stiffness, and topography markedly regulate cellular behavior [65]. The cortical region of subchondral bone is highly rigid, and its matrix is rich in hydroxyapatite [41, 66, 67]. Additionally, its trabeculae form a unique reticular scaffold structure, which may profoundly affect the activities of osteoblasts, osteocytes, and osteoclasts, thereby influencing OA progression (Fig. 2b). The synovium forms a barrier of synovial fibroblasts that separates synovial fluid from the underlying connective tissue, which is enriched in immune cells. Infiltration of immune cells into the synovium is a key driver of synovitis and OA-related pain (Fig. 2c) [68].

Finally, pronounced differences exist in the chemical microenvironments of the three tissues. Owing to its avascular nature, cartilage depends on diffusive nutrient transport from synovial fluid and therefore resides in a chronically hypoxic milieu; fluctuations in oxygen tension are pivotal regulators of type II collagen synthesis by chondrocytes [69, 70]. By contrast, both subchondral bone and synovium are highly vascularized and innervated, and vascular invasion into subchondral bone has been identified as an important event in OA progression [71]. The unique joint architecture allows cartilage and synovium to contact the same synovial fluid, while cartilage interfaces tightly with subchondral bone across the tidemark; nevertheless, each tissue preserves a distinctive chemical landscape (Fig. 2).

Overall, the central objective in constructing a JoC system lies in accurately replicating the physiological or pathological microenvironments of the joint. In this process, one can begin by modeling the three fundamental types of joint microenvironments summarized earlier. Building upon this foundation, specific research goals should guide the selection of one or more of these microenvironments as focal points, with corresponding experimental variables established to investigate how alterations in different microenvironments influence joint physiological function.

In summary, cartilage, subchondral bone, and synovium exhibit marked specificity in mechanical stimuli, structural attributes, and chemical microenvironments. A JoC must therefore recreate all three microenvironments within a single device, minimize undesirable cross-interference, and simultaneously permit physiologically relevant inter-tissue communication. The central challenge is the integration of appropriate mechanical stimulation with multicompartment tissue culture. The following sections survey JoC studies addressing mechanical loading and multi-tissue co-culture and discuss potential solutions to the design of mechanically stimulated, multi-tissue JoCs.

## Current Joint-on-a-Chip Systems

Current research on JoC platforms can be classified into three major categories: (i) devices that simulate only mechanical stimuli [72]; (ii) devices that simulate only multi-tissue co-culture [73]; and (iii) devices that simultaneously integrate mechanical stimulation with multi-tissue co-culture [74]. The following sections review each category and critically assess their respective strengths and limitations. Outstanding issues encountered during the integration of multi-tissue constructs with mechanical stimulation are summarized at the end.

### JoC with Mechanical Stimulation

Because joint tissues experience complex loading patterns, reproducing these biomechanical features on-chip remains challenging. To date, cartilage tissue is the most frequently investigated, and compression and shear are the two primary mechanical cues modeled. Accordingly, the discussion below focuses on strategies for applying compressive and shear forces to cartilage constructs within microfluidic platforms.

In contemporary chip designs, two overarching approaches are employed to deliver mechanical cues to engineered tissues. The first relies on fluid flow to generate shear stress at the cell surface; this strategy is widely used for tissues naturally exposed to body fluid flow, such as the vasculature [20] and kidney [17]. The second employs the deformation of polydimethylsiloxane (PDMS) under an external actuation force to impose tensile or compressive strain. Tensile loading is achieved by seeding the tissue on a PDMS membrane; deformation of this thin film stretches the resident cells. This mode of actuation originated in the conventional FlexCell [75] platform and has since been adopted in diverse in vitro physiological models, including lung [76], small intestine [77], and other OoC, exhibiting stretch behavior. The generation of compressive loads on-chip was introduced more recently. Analogous to tensile chips, the tissue construct is confined within a sealed chamber; deformation of the PDMS reduces the chamber volume, thereby exerting compression. Such designs are most prevalent for heart [19], skeletal muscle [77], and articular cartilage models that require cyclic compression.

**Fig. 3:**
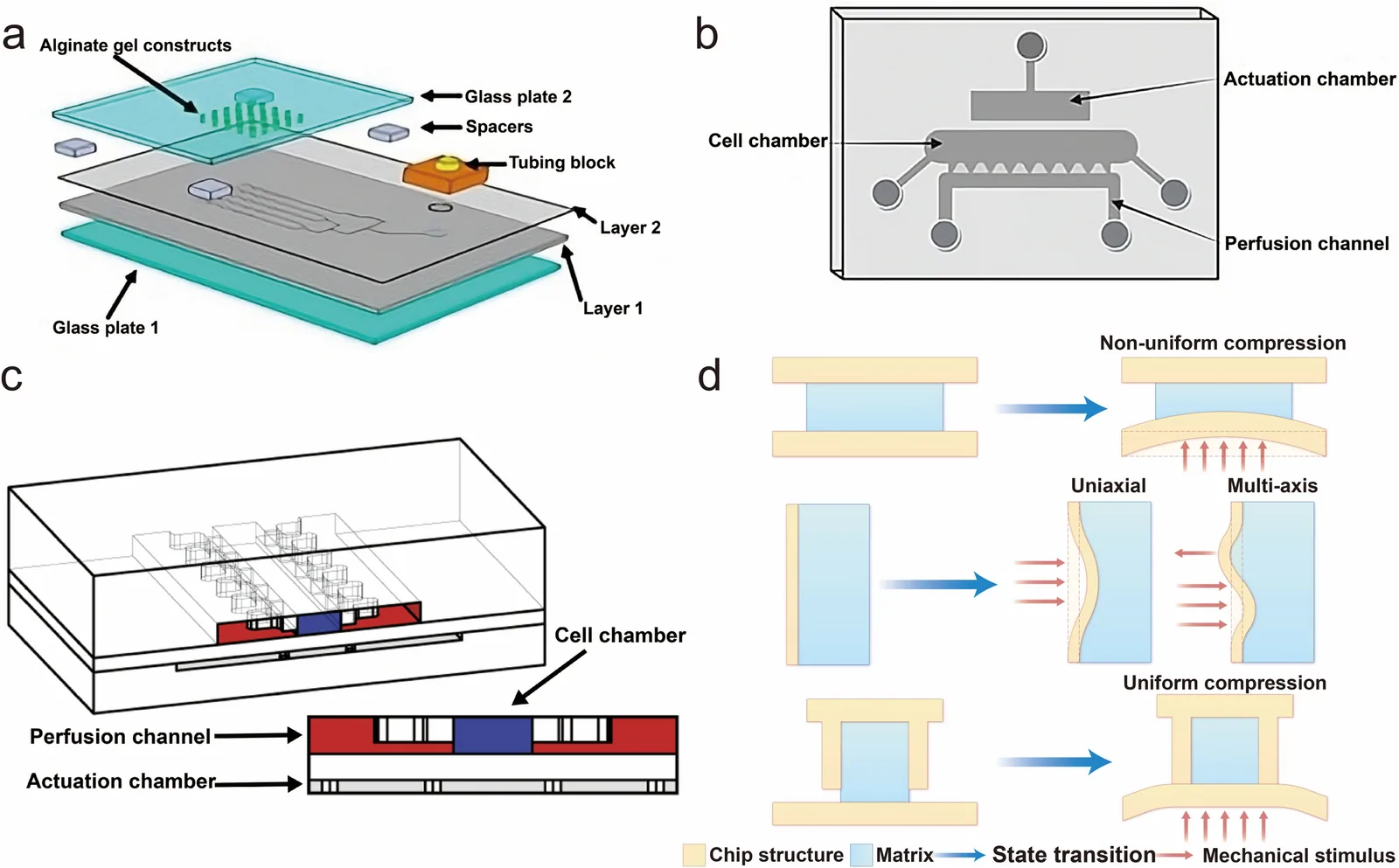
Schematic illustration of JoC models integrating various types of mechanical stimulation. **a** A high-throughput compression chip designed by Lee et al., featuring multiple airbags of different sizes that directly contact hydrogels via membrane vibration to generate localized mechanical stimulation. Reproduced with permission [78]. **b** A chip developed by Paggi et al. capable of applying multiaxial compression, equipped with an integrated multi-axis actuation unit. By applying positive or negative pressure to three independent chambers, multidirectional forces are exerted on the membrane, generating complex, and spatially heterogeneous mechanical stimuli. Reproduced with permission [80]. **c** A chip designed by Occhetta et al. for uniform compression, incorporating multiple micropillars spaced from the bottom surface. When the vibrating membrane contacts the pillars, it enables more stable and controlled uniform compression. The dimensions of the pillars can be adjusted to modulate the compression magnitude. Reproduced with permission [82]. **d** Schematic diagrams illustrating the mechanical stimulation mechanisms of chips **a**, **b**, and **c**, shown from top to bottom in corresponding order

Within joint research, PDMS deformation has been extensively explored to recreate the mechanical milieu of cartilage subjected to compression or shear. Lee [78] and co-workers were the first to apply a compression-generating OoC to cartilage (Fig. 3a). They used an array of PDMS pneumatic micro-balloons to apply graded compression to vertically oriented cartilage cell–alginate hydrogel micro-columns. The chip design enabled multiple compression ratios by controlling the balloon size, and a single device accommodated dozens of cartilage constructs, permitting high-throughput assessment of the cellular responses to various compressive strains. Nevertheless, the design has intrinsic limitations. Although compression ratios were regulated by pneumatic input and monitored microscopically, the inherent spherical deformation of the flexible PDMS under gas pressure resulted in a spatially heterogeneous stress distribution (Fig. 3d). Lateral imaging cannot resolve the local strain distribution within the construct; specifically, the hydrogel periphery experiences near-zero compression, whereas the central region is subjected to compressive strains substantially exceeding the preset value, thereby compromising experimental accuracy and reproducibility.

Paggi et al. designed a multiaxial compression chip that partially overcomes the shortcomings [79, 80]. By inflating PDMS pneumatic chambers with positive pressure, the device generates a gradient of strain ranging from physiological to supraphysiological levels, thereby mimicking the complex mechanical cues encountered by chondrocytes in vivo (Fig. 3b). When multiple balloons are integrated and individually actuated with positive or negative pressures, shear strain can also be imposed on the construct (Fig. 3d). Because the mechanical regimen can be programmed by adjusting the applied pressure and balloon geometry, the platform readily delivers diverse loading profiles. By applying physiological mechanical stimulation to cartilage, the authors successfully induced the formation of a more desirable cartilage phenotype. Moreover, under multidirectional mechanical loading, the PCM encapsulation formed more readily around chondrocytes, closely resembling that of native cartilage. Significant deposition of glycosaminoglycans (GAGs) and hyaline cartilage-associated proteins—including type II collagen, type VI collagen, and aggrecan—was observed around individual chondrocytes, resulting in the construction of chondron-like structures [81]. Unlike Lee’s vertically oriented micro-columns, Paggi et al. positioned the constructs as planar rectangular prisms, facilitating accurate microscopic quantification of local stress and displacement.

Nevertheless, the Paggi platform is not without limitations. Although the stress distribution within the construct can be mapped under multiaxial loading, regions experiencing specific mechanical stimuli cannot be isolated from the bulk tissue for downstream analyses such as cytokine secretion or gene expression. In addition, cells in zones with different strain magnitudes may communicate and thereby confound experimental interpretation. More critically, strain heterogeneity within the chip exceeds that observed in the native cartilage microenvironment. In human cartilage, although depth-dependent deformation varies considerably, the surrounding PCM damps these disparities, narrowing the range of strains experienced under physiological conditions. From both a mechanistic and translational perspective, imprecisely controlled loading regimens may therefore be suboptimal.

To achieve uniform and controllable compression, Occhetta introduced a chip architecture enabling confined compression [82]. The core component is an inverted rectangular chamber in which two parallel rows of T-shaped pillars are suspended from the ceiling, while the floor is spanned by a PDMS membrane (Fig. 3c). A fixed gap is maintained between the pillar tips and the membrane. After the chamber is filled with a cell-laden hydrogel and polymerized, pneumatic actuation deforms the membrane until it contacts the pillar bases, reducing the chamber volume by a predefined ratio and thereby compressing the tissue. Upon application of 10% physiological compression, the expression levels of cartilage-related genes (ACAN, PRG4, and COL2A1 relative to COL1A1) were comparable to those in healthy native cartilage, indicating that the system effectively recapitulates the physiological characteristics of cartilage. Meanwhile, the expression of cartilage homeostasis regulators FRZb and GREM1 was upregulated to native levels, suggesting that this model is progressing toward the maturation of a stable, articular cartilage-like tissue. In contrast, the application of 30% supraphysiological confined compression alone induced a catabolic imbalance, characterized by decreased ECM components (COL2A1 and ACAN), upregulated expression of IL6 and IL8, and a pronounced increase in MMP-13. These changes drove the cartilage from a homeostatic state toward an inflammatory and hypertrophic phenotype, exhibiting a gene expression profile closely resembling that of clinical OA samples. Compared with cytokine-based induction, this mechanically induced OA phenotype recapitulates human pathophysiology more faithfully and proves suitable for drug-testing applications. The principal advantage of this design lies in its ability to impose precisely defined, spatially uniform compression, facilitating rigorous parametric studies (Fig. 3d). However, the structure cannot deliver multiaxial compression or reproduce the shear forces experienced by superficial zone chondrocytes. Moreover, the device accommodates only a single tissue type and thus cannot emulate the integrated physiology or pathology of the whole joint.

The key to effective mechanical stimulation in JoC devices lies in precisely controlling both the loading modality and its magnitude. Current compression-control strategies fall into two categories: (i) pneumatic control and (ii) structural control. Pneumatic control modulates the applied pneumatic pressure and correlates the resulting deformation—visualized microscopically—with the desired compression ratio. Because PDMS is highly compliant, pressurization typically produces a spherical bulge whose curvature varies along its surface, generating pronounced spatial differences in tissue deformation and making the actual strain difficult to calibrate accurately [83]. Moreover, most constructs are embedded in hydrogels; post-polymerization swelling alters their mechanical properties, introducing additional deviation errors in the target strain [84].

Structural control addresses these issues by incorporating internal features that constrain PDMS to a fixed deformation ratio within a prescribed pressure window, thereby yielding uniformly distributed compression. The trade-off is increased fabrication complexity and reduced flexibility for multi-tissue culture. Incorporating several tissue types on a single chip while applying tissue-specific mechanical cues markedly complicates the design. Owing to the need for precise isolation of mechanical stimuli, adjacent culture chambers must be spaced sufficiently apart, yet fluidic interconnects must still permit biochemical communication between tissues. Achieving this balance often necessitates serially linked chips or intricate on-chip networks of pneumatic chambers and microfluidic channels. These engineering challenges make the concurrent integration of multi-tissue culture and programmable mechanical stimulation exceedingly difficult.

### JoC with Multi-tissue Cultivation

OA is a complex whole-joint disease, making the integration of multiple tissues within a single chip highly significant for studying joint disorders. Various designs have been developed to simulate the pathophysiological processes of osteochondral tissue, synovial, cartilage, and other related tissues (e.g., IPFP) in vitro.

**Fig. 4:**
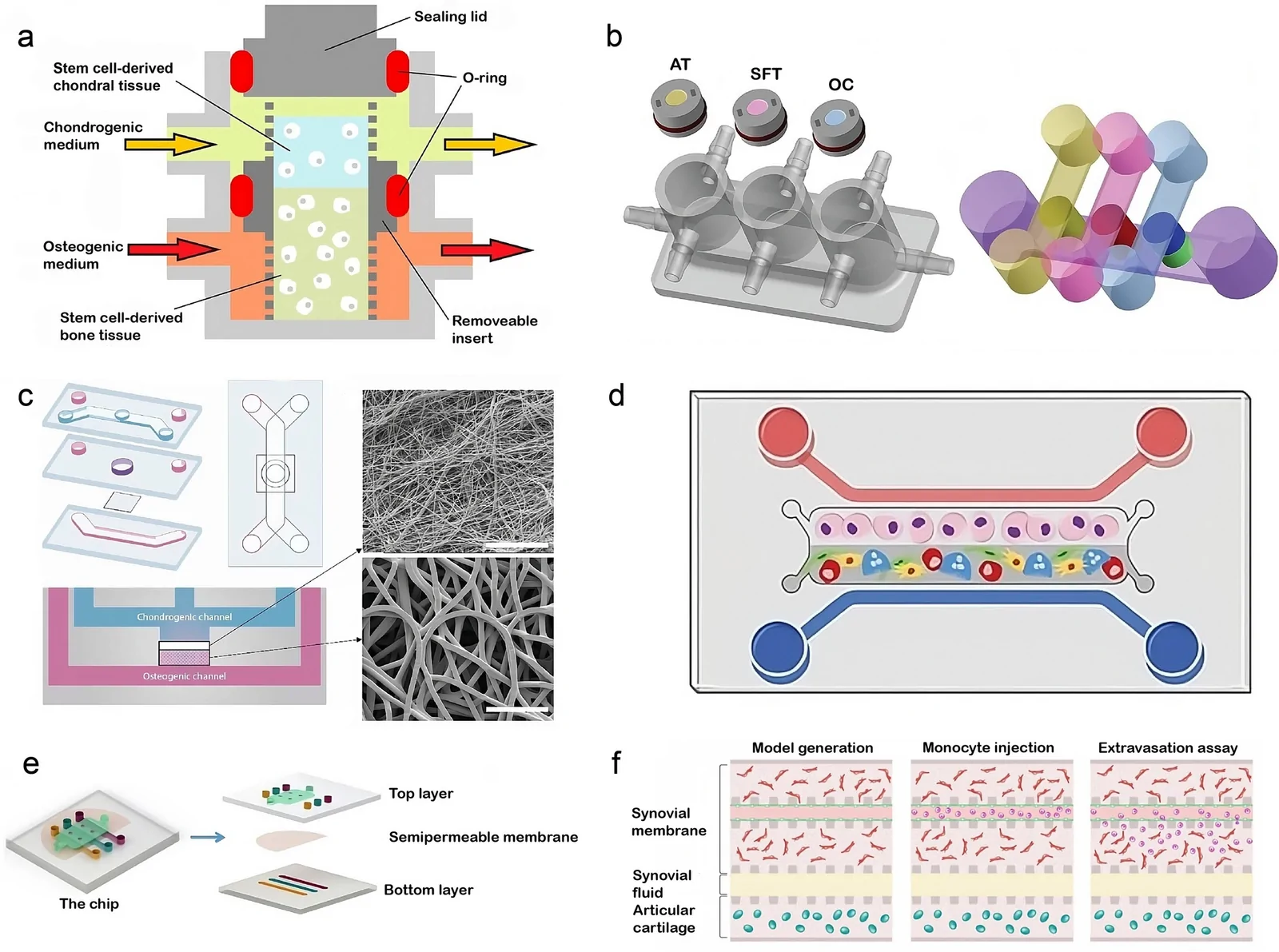
Schematic illustration of representative JoC systems incorporating multiple joint tissues. **a–d** Multi-tissue co-culture JoC models: **a** Chip containing an engineered osteochondral unit. Distinct fluidic channels supply tissue-specific media to support the physiological functions of each compartment. Reproduced with permission [85]. **b** A modular expansion based on the chip in **a**, with tissue components interconnected via a manifold, resulting in a comprehensive JoC containing an osteochondral unit, adipose tissue, and synovial tissue. Reproduced with permission [86]. **c** A chip comprising cartilage and bone tissues, utilizing electrospun polycaprolactone (PCL) scaffolds with both microfiber and nanofiber layers. Reproduced with permission [87]. **d** A chip integrating chondrocytes, osteoblasts, osteoclasts, endothelial cells, and mesenchymal stem cells (MSCs). Separate lateral channels deliver distinct media for cartilage and bone, allowing accurate simulation of osteochondral interactions as well as physiological and pathological processes within subchondral bone. **e**, **f** JoC combining multi-tissue co-culture with mechanical stimulation. Reproduced with permission [88]. **e** A chip featuring three parallel rectangular microchambers in the lower layer and one microchamber in the upper layer, separated by a polycarbonate membrane. Reproduced with permission [89]. **f** A chip partitioned into five chambers by pillar structures to simulate cartilage and synovial tissues. Chondrocytes, synovial fibroblasts, and endothelial cells are seeded in separate chambers, while monocytes are suspended within the medium. Controlled fluid flow imposes shear stress. Reproduced with permission [90]

In 2014, Lin et al. developed an osteochondral microphysiological bioreactor [85]. The bioreactor is cylindrical in shape and employs photopolymerization technology to layer hydrogel solutions containing different cell types within the chamber. By precisely controlling the solution volume and base area, chondrocytes can be seeded in the upper chamber and osteoblasts in the lower chamber. The two chambers are connected through a hydrogel interface, enabling direct cell–cell interactions between the two cell populations. By precisely controlling the placement of perfusion inlets for culture media, the system enabled in situ formation and co-culture of both bone and cartilage tissues while maintaining their respective phenotypes (Fig. 4a). To model OA-like joint degeneration, Lin applied interleukin-1β (IL-1β) to the cartilage or bone tissue. The results revealed that IL-1β stimulation of bone tissue triggered a more severe inflammatory response in cartilage tissue than directly insulting the cartilage by IL-1β, highlighting active biochemical signaling at the osteochondral interface.

Building on this foundation, Lin’s group further integrated multiple microphysiological dual-flow bioreactors into a single 3D-printed microfluidic bioreactor to enable the crosstalk between engineered osteochondral complex, synovial-like fibrous tissue, and adipose tissue [86]. Each tissue compartment receives its specific culture medium through independent channels. The shared culture medium in the bottom channel mediated the crosstalk between the tissue components (Fig. 4b). Each tissue compartment receives its specific culture medium through independent channels. The modular design can, in principle, accommodate any desirable number of tissues and reveal the role of any particular tissue component in joint pathogenesis. However, its main limitations include the absence of mechanical stimulation and the intricate microstructure of subchondral bone.

To better emulate the ECM of osteochondral structures, Tuerlings introduced a fibrous polycaprolactone matrix into the chip, seeding it with chondrocytes and osteoblasts (Fig. 4c). The microfiber layer was designed to mimic the bone matrix and support osteoblasts, while the nanofiber layer served as a separation interface between chondrocytes and osteoblasts, enabling precise control over cellular distribution and interfacial interactions. Upon establishing the co-culture system, deposition of cartilage matrix was observed within the chondrogenic compartment, whereas bone-like matrix formation occurred within the fibrous interlayer. When the resulting bone and cartilage tissues were exposed to active thyroid hormone, the expression levels of hypertrophic markers—integrin-binding sialoprotein and alkaline phosphatase—were significantly elevated, confirming that this system successfully recapitulates an OA disease model. This design aids in studying the repair processes of tissues following injury. Moreover, for modeling subchondral bone, osteoblasts alone are insufficient to replicate the entire physiological environment [87].

To address this, Salehi embedded chondrocytes in fibrin hydrogels and encapsulated osteoblasts, osteoclasts, endothelial cells, and mesenchymal stem cells (MSCs) in fibrin hydrogels enriched with calcium phosphate nanoparticles (Fig. 4d). By applying IL-1β to induce OA-like conditions, they combined cartilage and bone compartments to capture the complex pathophysiology of OA [88]. This multi-cellular approach more accurately simulates the response of subchondral bone during OA progression, with the inclusion of endothelial cells potentially recreating the phenomenon of vascular invasion into cartilage. Undoubtedly, incorporating more cell types into a single chip offers the potential to model the joint environment and OA progression with greater precision. However, the lack of synovial tissue and mechanical stimulation remains a limitation in the development of such in vitro models.

### JoC with Both Multi-tissue Cultivation and Mechanical Stimulation

In JoC research, some designs have attempted to integrate multi-tissue culture and mechanical stimulation, particularly fluid shear stress. The first report implementing controlled fluid shear stress in a JoC appeared in 2017. Bao et al. engineered a two-tier microchamber device in which the bottom tier contained three parallel, vertically oriented microchambers and the top tier a single chamber; the two tiers were separated by a porous polycarbonate (PC) membrane [89]. Scaffold-free monolayers were seeded on the chamber walls, and a constant shear stress was generated by perfusing medium through the bottom tier. Chondrocytes, chondrogenically induced MSCs, or their mixtures were cultivated in the bottom chambers, whereas osteogenically induced MSCs occupied the top chamber (Fig. 4e). Fluid shear stress in the lower chamber was employed to simulate the hydrodynamic environment experienced by cartilage tissue, while the upper chamber contained osteogenically induced MSCs to reproduce the dynamic interactions between cartilage and subchondral bone. Using this chip-based platform, the authors systematically investigated how chondroinduced MSCs, chondrocytes, and their co-culture systems influence the morphological changes, proliferation rate, and phenotypic responses of osteogenically induced MSCs under mechanical microenvironmental conditions. The results demonstrated that chondrocytes and chondroinduced MSCs elicited similar responses in osteogenically induced MSCs. Moreover, the dedifferentiation effect of fluid shear stress on chondrocytes could be counteracted by stimulation from osteogenically induced MSCs, underscoring their crucial role in maintaining chondrocyte phenotypic stability. The combination of shear stress and osteogenically induced MSCs synergistically maintained a stable chondrocyte phenotype. However, this design neither achieved 3D tissue culture nor provided compressive mechanical stimulation for cartilage.

Synovial inflammation is closely associated with the onset and progression of OA and may occur in both early and late stages of the disease. Synovial cells trigger and sustain inflammation by regulating the secretion of inflammatory mediators, thereby damaging cartilage. To study synovial inflammation, Mondadori et al. developed a five-channel structure separated by four rows of dense micropillars. Individual channels were populated with synovial fibroblasts, endothelial cells, macrophages, and chondrocytes to model the extravasation of macrophages across the endothelium observed in OA (Fig. 4f). Furthermore, endothelial cells were activated by combined fluid shear stress and TNF-α stimulation to better mimic the synovial inflammatory environment [90]. This design successfully recapitulated the pathological activities of synovium in OA and accurately modeled the relationship between synovium and cartilage through the integration of shear stress and synovial endothelial cells. However, for cartilage and subchondral bone tissues, most affected by mechanical stimulation, the design failed to provide relevant mechanical stimuli, posing a significant limitation for OA research.

In summary, while existing designs have made progress in integrating fluid shear stress with cartilage or synovial endothelial cells, they have yet to comprehensively replicate the critical microenvironment of JoC. For instance, in cases where fluid shear stress was combined with cartilage, the lack of a 3D culture environment was evident. Conversely, when fluid shear stress was applied to synovial endothelial cells, cartilage and subchondral bone, along with their associated mechanical stimuli, were excluded. These limitations hinder the ability of JoC models to fully mimic the physiological and pathological environment of joints.

## Ideal JoCs: Requirements, Challenges, and a Proposed Prototype

The core of a JoC is to recreate the joint microenvironment as faithfully as possible within the microfluidic platform. However, most existing JoC models can only reproduce one facet of the joint microenvironment—such as mechanical stimulation or multi-tissue interaction—within an individual chip. Although some designs have attempted to combine these two aspects, their key functional elements have yet to be fully integrated. Accordingly, this chapter summarizes the design requirements, current challenges, and potential solutions for developing an ideal JoC, based on the unique characteristics of the joint microenvironment. Furthermore, a prototype design for a JoC is proposed.

### Design Requirements for Chip

The construction of an ideal JoC requires careful consideration of multiple factors, including tissue diversity, specificity of mechanical stimulation, and precise control of the chemical microenvironment. Despite recent advancements in the design of JoC, significant challenges remain. This section discusses the critical issues and potential approaches for constructing an optimal JoC.

Firstly, an ideal JoC must include at least three key tissues: cartilage, subchondral bone, and synovium. Additionally, the diverse cell types present in subchondral bone and synovium must be accurately simulated to meet various research needs. For multi-tissue culture designs, the chip requires multiple independent chambers, with each chamber dedicated to the culture of a specific tissue. To study the layered structure of cartilage, at least two to three chambers are needed to simulate the responses of the superficial, middle, and deep layers to different mechanical stimuli [91]. The synovium chamber should incorporate channels to mimic endothelial barriers, blood flow, and immune cell extravasation [92]. For subchondral bone, chamber designs must be further refined, utilizing 3D printing technologies to create hydroxyapatite-rich scaffolds that replicate the microstructural features of the SBP and trabeculae [93–95]. In particularly, simulating the specific microstructures of subchondral bone may require multiple chambers [96]. Furthermore, the culture of different tissues demands specialized media, such as osteogenic differentiation media, hypoxic media, or media tailored for specific drug studies [97]. The spatial arrangement of chambers should also be optimized to facilitate interactions between cartilage and subchondral bone, as well as between cartilage and synovium. Ultimately, a universal channel design is needed to simulate the flow of synovial fluid.

Secondly, addressing the need for mechanical stimulation, the chip must apply specific mechanical forces to the tissues within each chamber, including compression and shear forces for cartilage, micro-compression for subchondral bone, and shear stress from fluid flow for endothelial cells in the synovium. The compressive and shear forces experienced by chondrocytes represent the primary mechanical environments of the superficial and deep layers of cartilage. When chondrocytes are embedded within hydrogel scaffolds, the processes of water expulsion during compression or shear can partially simulate fluid shear or hydrostatic pressure effects, although these simulations remain imprecise and difficult to fully control [98]. The key to applying specific mechanical stimulation lies in strictly confining the mechanical forces to the target tissue without affecting adjacent tissues.

In summary, the comprehensive design of a JoC must balance mechanical stimulation and multi-tissue culture. On one hand, the chip must accommodate more joint-related tissues while providing them with specific chemical and biomechanical microenvironments. On the other hand, it must enable precise control over the intensity of mechanical stimulation while simultaneously supporting multi-tissue co-culture and establishing communication pathways between tissues. However, no study to date has successfully integrated these two aspects, primarily due to the design complexity arising from their combined requirements. Specifically, in the control of mechanical stimulation, existing designs such as the pillar structure developed by Occhetta—though capable of adjusting compression ratios via height modulation—face two major issues: (1) compression is difficult to confine to specific regions and may uncontrollably affect adjacent areas, and (2) when designing multiple chambers to house different tissues, the chemical microenvironment of each chamber must be independently considered, such as the hypoxic environment for cartilage, the mineralized microenvironment for subchondral bone, and specialized microenvironments for drug research [82, 99]. The need to simultaneously provide each tissue with a unique mechanical and chemical microenvironment while enabling inter-tissue communication imposes stringent requirements on-chip design. Simplifying chip design to achieve precise control of mechanical and chemical microenvironments remains the key challenge in constructing an ideal JoC [100].

### Mechanical Stimulation Control Challenges

The design approach for mechanical stimulation control can focus on optimizing control methods to accommodate the requirements of multi-tissue co-culture, while simultaneously simplifying the complexity of chip structures through innovative nutrient supply strategies. The ultimate goal is to develop a fully functional and practical JoC. The core challenge in mechanical stimulation control lies in the precise regulation of deformation ranges. The current technical bottleneck is the lack of suitable structures to constrain deformation within limited spaces. This issue primarily stems from the inertia of current chip fabrication techniques.

At present, OoC manufacturing typically relies on photolithography to achieve sub-micrometer resolution [101]. Despite its high precision, photolithography remains cost-prohibitive. Moreover, the primary limitation of conventional photolithography lies in its ability to generate only upright, high-aspect-ratio micro/nano structures [102]. These vertical sidewall structures lack 3D features, thereby limiting functionality. In contrast, 3D printing and machining technologies, though slightly less precise, enable the creation of gradient sloped and curved structures with adjustable angles [103]. These 3D features are key to addressing deformation control challenges.

Specifically, 3D printing or machining techniques can be employed to design the following structure: a concave circular chamber is formed on a PDMS membrane, with an array of pillars arranged in an inner ring within the chamber. These pillars are designed with specialized 3D geometries, where the top surface consists of two horizontal planes and one sloped plane. One horizontal plane is flush with the chamber’s top surface, while the other horizontal plane is offset by a certain height difference. The sloped plane connects the two horizontal planes, forming a continuous stepped structure (Fig. 6a). During chip operation, the circular chamber is covered with a rigid flat plane, and dynamic stimulation is applied to the chamber’s bottom, causing its base to deform into a convex shape. Due to the stepped structure of the pillars, the sloped face first contacts the chamber ceiling, the first horizontal plane then rapidly adheres to it, and finally, constrained by the second horizontal plane, the chamber’s deformation is precisely reduced to zero at that second plane. Furthermore, by arranging pillars of varying heights within the chamber, coexisting compressive and shear stimulation can be achieved (Fig. 6b).

### Reducing the Complexity of Multi-Tissue Chips

The complexity of chip design primarily arises from the need for biomimetic 3D architecture, which significantly increases the intricacy of channel design within 2D spaces. As OoCs are mostly established with microfluidic chips, channel designs from microfluidic systems are often adapted to supply nutrients to tissues [104]. However, there is an essential distinction between the two: microfluidic chips are fluid-centric, whereas OoC are tissue-centric, with fluid serving only as an auxiliary function [105]. Thus, channels are not the only option for designing nutrient delivery systems.

By innovating nutrient supply strategies and combining them with traditional channels, the complexity of chip design can be significantly reduced while offering greater design flexibility for tissues that require channel-based perfusion. Surface-based permeation is a promising approach for nutrient delivery. Its fundamental principle involves spatially segregating tissue blocks and culture medium into different levels within a 3D space, enabling continuous nutrient supply through interconnected planar channels.

**Fig. 5:**
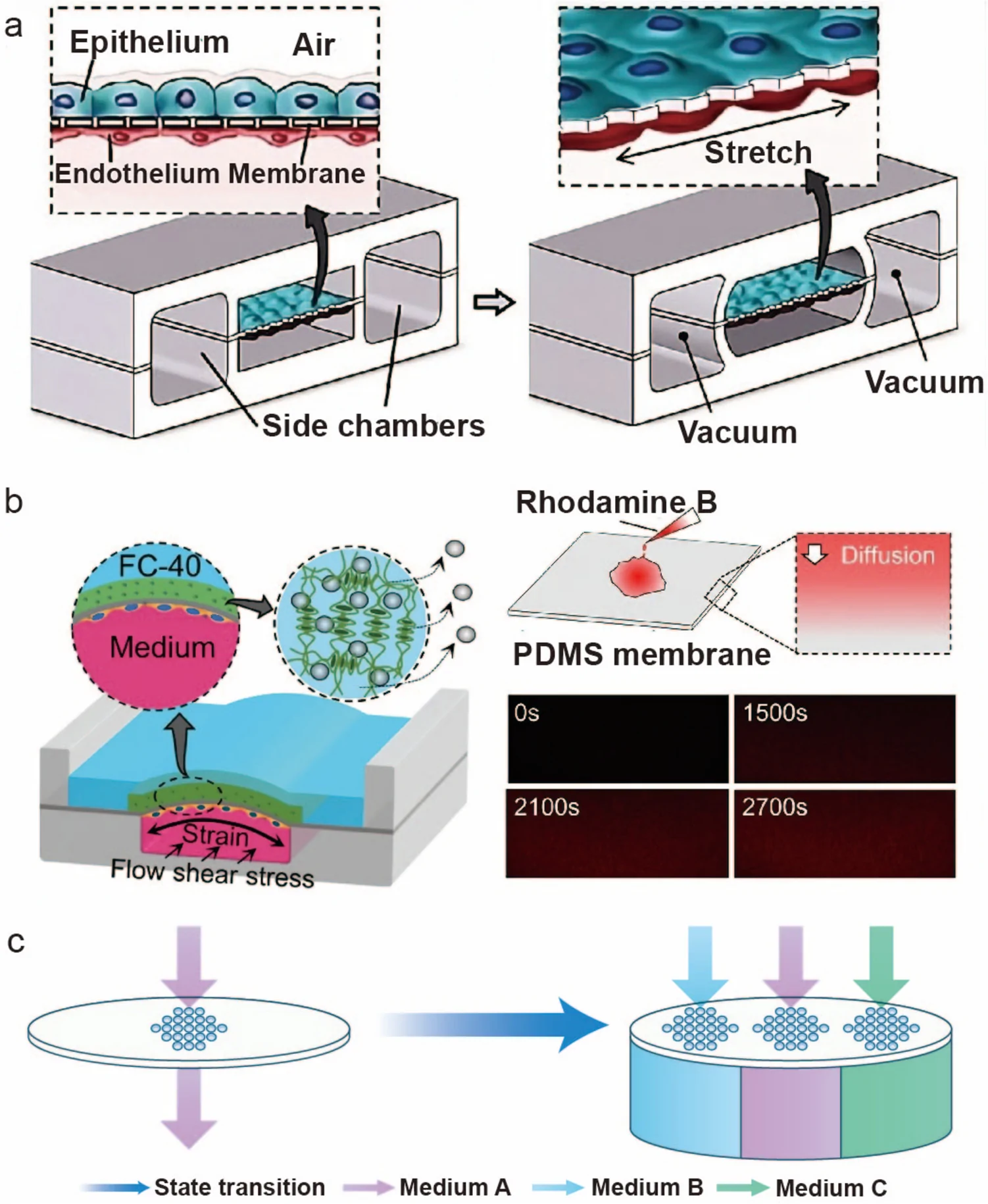
Schematic illustration of OoC designs based on various membrane surface-based permeation strategies. **a** A classic lung-on-a-chip developed by Ingber et al., featuring a porous elastic membrane seeded with cells on both sides. One side is exposed to culture medium, while the other is exposed to an air interface, simulating the air–liquid interface culture of pulmonary epithelial cells. Cells on the air-exposed side receive nutrients via transmembrane permeation. Reproduced with permission [11]. **b** On the left side of the figure, a dual-interface microfluidic chip developed by Fan et al. is shown, in which drugs are introduced on one side of a PDMS membrane, allowing lateral permeation into the membrane and continuous delivery to cells on the opposite side, enabling efficient localized drug administration. On the right side of the figure, visualization of drug permeation behavior in the PDMS membrane using Rhodamine B dye is presented to verify its permeation kinetics and distribution patterns. Reproduced with permission [94]. **c** Schematic of surface-based permeation principles, illustrating a strategy for precise nutrient delivery and microenvironmental modulation to underlying tissues via a single membrane with specifically designed pore sizes and arrangements. This approach enables the provision of tissue-specific media to different cell types simultaneously

Since Ingber’s design of the first lung-on-a-chip, surface-based permeation techniques have emerged [13]. This method typically employs a porous membrane seeded with cells on both sides, with culture medium delivered via planar channels (Fig. 5a). This design rapidly expanded to various tissue chips, including intestinal and vascular systems. Fan et al. designed a dual-interface microfluidic device in which drug-loaded alginate hydrogel sheets were adhered to a PDMS membrane, enabling gradual drug release and precise delivery to cell monolayers (Fig. 5b) [106]. This innovation not only addressed the issue of PDMS adsorption of hydrophobic drugs but also achieved targeted drug administration for specific tissues.

However, whether in Ingber’s classic lung-on-a-chip or Fan’s innovative design, cell culture in these chips is typically limited to a quasi-3D (2.5D) monolayer format. In chips employing 3D culture, surface-based permeation designs are less commonly utilized. This is because gel blocks or scaffolds are inherently 3D structures, and nutrient delivery can generally be achieved via channel integration. Adding a chamber on another surface would only increase design complexity. Nevertheless, surface-based permeation technology retains unique advantages. First, as an extension of channel-based designs, surface-based permeation can provide more comprehensive nutrient delivery, addressing the diffusion limit of traditional designs and enabling greater flexibility in tissue block shapes and volumes. Additionally, its 3D characteristics make it a complementary method to channel-based nutrient delivery systems, offering enhanced compatibility. Surface-based permeation can deliver specific culture media to different tissues at any location, thus resolving the conflict in multi-tissue co-culture systems where distinct media are required while maintaining inter-tissue communication (Fig. 5c). Consequently, surface-based permeation plays a critical role in the construction of JoC, significantly reducing the complexity of joint design.

### A Proposed JoC Prototype

Based on the resolution of the two key challenges, a prototype of a JoC integrating multi-tissue co-culture and specific mechanical stimulation can be designed. Specifically, the proposed structural scheme for the design and construction of the JoC is as follows: A multilevel structure is built using continuous stepped structural pillars, with a single pillar positioned on the far-left side, while the remaining pillars gradually increase in height from left to right. Within the chambers enclosed by these pillars, multi-layered cartilage is placed in the left chambers to simulate the superficial, middle, and deep layers of cartilage, respectively. The variations in pillar height produce different types of mechanical stimulation: high-shear strain with low compressive strain simulates the superficial and middle cartilage layers, while high compressive strain simulates the deep cartilage layer. Specifically, in the central and external regions of the chip, gradient pillars of varying heights are arranged sequentially. First, a series of micropillars with varying heights are arranged at the center to mimic the superficial cartilage layer, which is characterized by a high-shear, low-compression mechanical environment. Subsequently, a combination of stepped structural pillars and standard micropillars is employed to reproduce the high-compression conditions typical of the deep cartilage layer. The sequential alignment of the two stepped structural pillars is designed to emulate the mild mechanical stimulation experienced by the subchondral bone region, while the terminal section is conFig.d to achieve a non-compressive state, thereby replicating the environment of the synovial tissue.

**Fig. 6:**
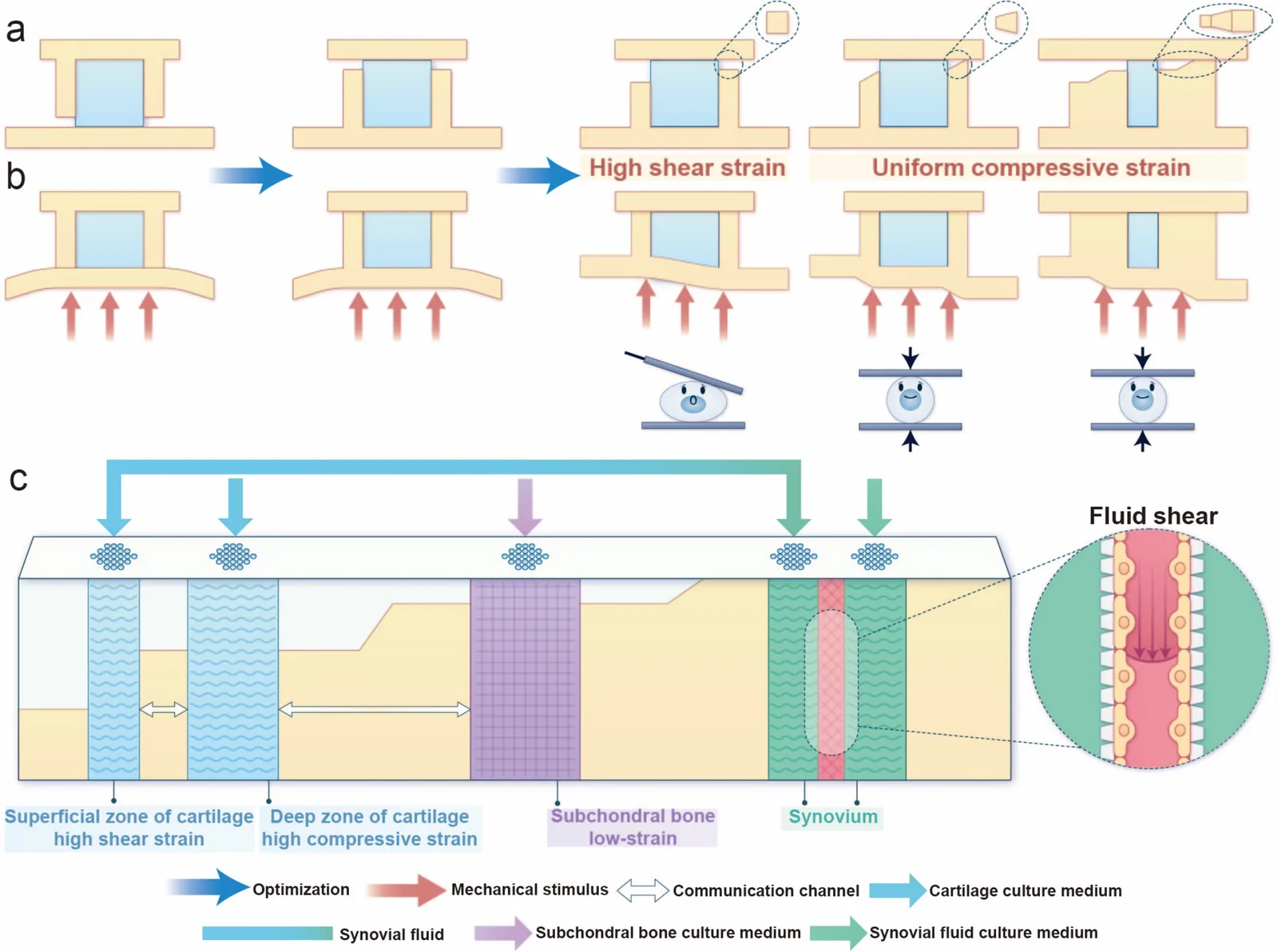
Schematic illustration of a multi-tissue JoC design integrating stepped structural pillars and surface-based permeation strategies. **a** Conceptual evolution of the stepped structural pillars design. From left to right: the leftmost image shows the original design proposed by Occhetta, enabling uniform compression of a single tissue; the second step relocates the micropillars beneath the membrane and uses the upper surface as a permeable interface, laying the foundation for combining mechanical stimulation with nutrient delivery; further optimizations include the design of micropillars with height variations (third), inclined micropillars (fourth), and finally stepped structural pillars (fifth), enabling more complex simulation of mechanical microenvironments. **b** Schematic of the types of mechanical stimuli generated by different chip structures. These chip architectures can provide two types of mechanical stimulation: uniform compression and shear stress. Their structural design allows precise control of these microenvironments within a single chip. **c** Integrated multi-tissue JoC schematic based on stepped structural pillars and surface-based permeation principles. By integrating the two design concepts of stepped structural pillars and surface-based permeation, this approach enables precise control of mechanical stimulation through the stepped structural pillars, while the surface-based permeation mechanism provides a tissue-specific chemical microenvironment and simplifies the overall chip architecture

In addition, chambers separated by micropillars enable the simulation of the complex architecture of the synovium, including the formation of vascular-like structures following endothelial cell seeding and the generation of fluid shear stress resulting from medium perfusion. The subchondral bone chamber incorporates a 3D-printed scaffold designed to recapitulate the bone microarchitecture, thereby influencing cellular behavior through structural cues. Beyond precise control of the mechanical microenvironment, the surface-based permeation strategy allows the delivery of tissue-specific culture media to the cartilage, subchondral bone, and synovium, faithfully recreating their respective biochemical milieus. Notably, the partial interconnection between the cartilage and synovial media serves to mimic the physiological function of synovial fluid. Furthermore, the interstitial gaps formed between the micropillars—linking the superficial and deep cartilage chambers, as well as the deep cartilage and subchondral bone chambers—facilitate the exchange of molecules and signaling factors, supporting interactions both within cartilage zones and between cartilage and subchondral bone. To reconstruct the continuous in vivo interfaces of these tissues on-chip, surface functionalization can be applied to specific regions to maximize inter-pillar spacing, while the fluidic properties of hydrogels permit intimate and continuous contact between adjacent tissues, thereby enabling extensive cross-tissue interactions (Fig. 6c).

Above this planar structure, multiple specially shaped chambers are designed, with shapes and sizes corresponding to the underlying chambers. These chambers can simulate the unique chemical microenvironment of different tissues by infusing specific culture media, providing nutrients and communication pathways to the underlying tissues via surface-based permeation (Fig. 6c).

The incorporation of continuous stepped structures and surface-based permeation structures addresses two key challenges in the design of the JoC: the integration of mechanical stimulation control with multi-tissue co-culture. Specifically, the design of continuous stepped structural pillars allows for the application of controllable, specific mechanical stimulation exclusively to cartilage and subchondral bone, without interfering with other tissues. The surface-based permeation structure, on the other hand, provides theoretical support for implanting multiple tissues within a single chip while facilitating their functional communication.

The combination of these two structures offers significant advantages: multiple tissues can be arranged on the same plane based on research requirements, with pillar designs enabling the delivery of tissue-specific mechanical stimulation. Additionally, the structures allow for communication between tissues and the supply of specific culture media, thereby simulating their chemical microenvironments. The resulting chip prototype can accommodate multiple joint-related tissues, including cartilage, subchondral bone, and synovium, accurately reproducing their respective mechanical and chemical microenvironments, and offering inter-tissue communication channels as required. Building on this foundation, researchers can introduce various joint tissues as needed, providing them with appropriate biomechanical and chemical microenvironments to ultimately establish a JoC applicable to diverse research fields. For example, osteal macrophages (OsteoMacs) represent a resident macrophage subpopulation specifically located within bone tissue, playing a critical role in maintaining bone homeostasis [107]. However, their precise biological functions have not yet been fully elucidated. Therefore, based on the chip prototype established in this study, a more specific in vitro model targeting OsteoMacs can be further developed to facilitate in-depth investigations into their functional mechanisms.

## Outlook

### Commercialization of JoC

As research into JoC systems continues to advance, several high-performance, commercially available microphysiological platforms have already entered the market. For example, BiomimX’s uBeat™ platform recreates human tissues’ biomechanical behaviors, whereas Chiron has developed JoC models for simulating joint pathologies. Nevertheless, despite considerable progress toward commercialization, the large-scale implementation of JoC devices in clinical practice and drug development pipelines still encounters numerous hurdles. Key issues include standardization and modular design, cell sourcing, detection, and analytical methods.

Standardization and modular design are critical for JoC. Currently, various sophisticated designs have been proposed to simulate the joint microenvironment. However, the differing requirements of clinical and drug development applications may demand chips with specific focuses, such as synovial inflammation, osteophyte formation, or cartilage degradation. To reduce costs and avoid redundant designs, it is necessary to establish standardized functional modules and enable their rapid assembly and application through a unified framework [108]. This on-demand customization and rapid integration process will accelerate the commercialization of JoC. For instance, the Giselbrecht team developed a modular chip with a plug-and-play assembly akin to LEGO blocks, allowing for the integration of multiple tissues with high scalability [109]. However, this approach has limitations in precisely controlling the microenvironment. Another strategy involves establishing a standardized platform for JoC that integrates a library of structures essential for simulating the joint microenvironment. Researchers can use these standardized structures to develop JoC tailored to their specific needs. This approach not only minimizes waste from redundant designs but also significantly accelerates research and commercialization processes. The JoC prototype proposed in this study, featuring designs such as stepped structural pillars and surface-based permeation, can simulate most aspects of the joint microenvironment. Among them, stepped structural pillars and surface-based permeation structures, owing to their excellent scalability, enable flexible definition of mechanical stimulation microenvironments and multi-tissue co-culture configurations within a certain range. Therefore, this design can be adapted by different research groups as needed during the development of JoC systems, gradually evolving into a standardized foundational module. It also has the potential to integrate existing single-chip designs, laying a foundation for the development of a universal platform in the future.

Cell sourcing is one of the primary obstacles to the commercialization of JoC. Currently, most chips utilize human primary cells. However, the non-proliferative nature of chondrocytes makes the acquisition of primary chondrocytes expensive and resource-limited. Inducing the differentiation of bone marrow mesenchymal stem cells into articular cartilage is a potential solution, as these stem cells can be isolated from various tissues and expanded on a large-scale in vitro, thereby minimizing donor dependency [110]. Another promising cell source is induced pluripotent stem cells (iPSCs), which are generated by reprogramming somatic cells, thus avoiding the need for primary cells from multiple tissues [111, 112]. Theoretically, the unlimited expansion capacity of iPSCs makes them ideal for high-throughput drug screening applications in OoC systems. However, challenges remain regarding the maturity, operational complexity, and cost of iPSC-derived cells, necessitating further optimization before commercial application [111]. A third alternative is the use of animal-derived primary chondrocytes [113]. Despite species differences, these cells are cost-effective and readily available. Compared to traditional animal experiments, JoC can provide specific microenvironments tailored to research needs, which is significant for OA studies. This approach also substantially reduces the use of experimental animals, shortens research timelines, lowers costs, and alleviates ethical concerns. Joint chips integrating animal cells can reduce expenses and serve as intermediate tools to replace animal experiments, making them more accessible for drug development by enterprises.

Detection methods for JoC should be as compatible as possible with existing laboratory techniques and data acquisition equipment to facilitate widespread adoption by researchers [114]. However, due to the technical limitations of chips themselves, developing detection methods specifically adapted for chips is equally important. OoC systems typically cultivate the minimal functional unit, often producing trace amounts of output that may not be compatible with existing detection methods. Some researchers have integrated biosensors into chips to enable real-time monitoring of multiple biochemical indicators [115, 116]. This type of wash-free detection technology allows for direct analysis of trace samples on the chip [117]. Additionally, microfluidic chips, as a next-generation technology for precise detection with low sample consumption, have been developed for various biological analyses, including nucleic acids [118] and proteins [119, 120]. The integration of microfluidic technology with OoC systems can meet the high-throughput detection requirements for trace outputs, holding significant potential for the commercialization of OoC applications.

### Multi-organ-on-a-Chip

In the future, the JoC platform should be integrated with other OoC systems to construct multi-organ-on-a-chip (multi-OoC), enabling deeper investigation into the mechanisms and therapeutic strategies of joint diseases. Multi-OoC plays a crucial role in studying inter-organ interactions and signal crosstalk [121]. Compared with single-organ culture systems, multi-organ platforms provide a more comprehensive approach for assessing drug safety and efficacy [122]. For instance, epidemiological studies have shown that women are more susceptible to OA [123]. A combined joint–ovary chip could therefore be utilized to explore the role of estrogen in OA pathogenesis. Moreover, there is increasing evidence of a close association between OA and pulmonary diseases. Studies have reported that individuals with knee or spinal OA exhibit reduced lung function compared with non-OA subjects, which may be linked to shared pathogenic mechanisms involving chronic inflammation [124]. Thus, constructing a joint–lung integrated chip would provide a powerful platform for elucidating these interrelated disease processes. Finally, in the context of drug development, toxicological assessment remains a critical step. By coupling a liver-on-a-chip with the JoC, researchers can investigate the hepatically metabolized toxicity of OA-related drugs, thereby providing more reliable and physiologically relevant data for drug safety evaluation [122].

### Policy Development

Beyond technological advances, policy orientation is also a critical factor determining whether JoC systems can be widely adopted by research institutions and pharmaceutical companies. As societal demands for more physiologically relevant disease models continue to rise, OoC technologies have increasingly attracted the attention of regulatory agencies. As early as 2010, the European Union promoted the “3R” principles (Replacement, Reduction, and Refinement of animal experimentation), encouraging the use of cell-based approaches to minimize animal testing [125]. In recent years, the U.S. Food and Drug Administration (FDA) has consistently supported the application of organoids and OoC systems, proposing the gradual phase-out of animal testing and the incorporation of microphysiological systems into the non-clinical drug evaluation framework [126]. In July 2025, the National Institutes of Health (NIH) announced that it would cease funding research projects relying solely on animal experiments. Similarly, in 2021, China included organoid research within its National Key R&D Program. Overall, policy support for OoC technologies is steadily increasing across various countries, and the investment and expectations from both society and research institutions are also rising. With the continuous improvement and standardization of related technologies, OoC are expected to become an effective, and potentially complete, alternative to animal experimental models in the future.

## Conclusion

This review first introduces the fundamental structures and functions of three tissues closely associated with OA: articular cartilage, subchondral bone, and synovium. Based on these insights, the study outlines the relevant microenvironment required for constructing JoC systems and identifies one of the core challenges in current JoC development—designing platforms that are suitable for multi-tissue co-culture and specific mechanical stimulation. Building on this, the study reviews existing types of JoCs that simulate multi-tissue co-culture and mechanical stimulation, summarizing two key issues related to chip integration: precise control of mechanical stimulation and optimization of chip design. To address these challenges, the study proposes solutions involving gradient pillars and surface permeability, while further conceptualizing an idealized JoC model. Overall, JoC, as emerging in vitro models of human joint diseases, hold significant potential for advancing mechanistic studies and drug development for OA treatment. However, their future commercialization and laboratory applications will require addressing several critical issues, including the establishment of complementary detection facilities, chip standardization, and the personalization of therapeutic protocols.
